# Cardiometabolic Status of Adults Living with HIV in Panama—Baseline Results of the Colón C3 Study

**DOI:** 10.3390/medsci14020200

**Published:** 2026-04-15

**Authors:** Humberto López Castillo, Lorna E. Jenkins S., Víctor Israel Peñafiel Medina

**Affiliations:** 1 Department of Health Sciences, College of Health Professions and Sciences, Academic Health Sciences Center, University of Central Florida, Orlando, FL 32816, USA; 2 Department of Population Health Sciences, College of Medicine, Academic Health Sciences Center, University of Central Florida, Orlando, FL 32827, USA; 3 College of Public Health, University of South Florida, Tampa, FL 33612, USA; 4 Jenkins-Lara Consulting, LLC, Panama City, Panama; 5 HIV and STI Program, Ministry of Health, Colón City, Panama

**Keywords:** metabolic syndrome, cardiometabolic disease, adults living with HIV, antiretroviral therapy, social determinants of health, prevalence, Panama

## Abstract

**Background.** Cardiometabolic diseases (CMDs) have become a major health concern among adults living with HIV (ALWH) as antiretroviral therapy (ART) extends life expectancy. Metabolic syndrome (MetS)—a cluster of abdominal obesity, hypertension, hyperglycemia, hypertriglyceridemia, and hypoalphalipoproteinemia—is a key predictor of CMD risk. Despite high HIV prevalence in Panama, data on MetS among ALWH are scarce. Thus, the Colón C3 Study aimed to estimate the prevalence of MetS and its criteria in a large cohort of ALWH in Colón, Panama. **Methods.** Between April–December 2024, 659 ALWH aged ≥18 years were enrolled at the province’s sole ART Clinic (78.1% of active patients). Participants completed a computer-assisted survey on demographics and social determinants of health (SDoH), underwent anthropometry and body composition assessment, and provided ≥8 h fasting blood samples for glucose, lipid profiles, HbA1c, and high-sensitivity C-reactive protein (hsCRP). MetS was defined using NCEP-R ATP-III criteria, and analyses were stratified by sex. **Results.** Mean age was 43.9 (range 18–79) years; 55% were female, and 51% identified as Black/Afro-Caribbean. The overall prevalence of MetS was 38.6% (binomial 95% CI 34.5%, 42.9%), exceeding pooled estimates for ALWH in the Americas (30.4%). Among individual criteria, hypoalphalipoproteinemia (59.6%) and hypertension (52.6%) were most prevalent, followed by abdominal obesity (45.2%), hyperglycemia (33.5%), and hypertriglyceridemia (22.5%). Women exhibited significantly higher body fat mass and BMI than men. Mean hsCRP was 7.2 mg/L, indicating persistent inflammation despite virologic suppression. Socioeconomic vulnerabilities, food insecurity (30%), and housing instability (>40%) were common. **Conclusions.** Findings reveal a substantial cardiometabolic burden among ALWH in Colón and underscore the need for integrated HIV–CMD care models, earlier screening, and natal sex–responsive interventions. The results provide foundational evidence for improving long-term, equitable cardiometabolic outcomes in HIV care across Panama and the broader Latin American region.

## 1. Introduction

Cardiometabolic diseases (CMDs) are among the leading causes of morbidity and mortality worldwide [1]. Among CMDs, metabolic syndrome (MetS) is a cluster of interrelated conditions (i.e., abdominal obesity, hypertension, hypertriglyceridemia, hypoalphalipoproteinemia, and hyperglycemia) that greatly contribute to the global burden of CMD morbidity and mortality [1], where MetS has reached hyperendemic levels, affecting approximately one-quarter of adults [1,2] and has contributed to losses of quality of life, increased disability, and premature death [3]. In Latin America, the prevalence rates of MetS range from 19% to 43%, with Latin American adults exhibiting an age-standardized prevalence of 40% [4]. Panama mirrors these trends, with high rates of hypertension (46.5% in men and 42.1% in women), obesity (12.6%), and type 2 diabetes mellitus (DM2; 12.4%) [5,6,7,8,9,10]. Notably, CMD is the leading cause of death in Panama, accounting for 54.9% of all deaths in 2023 [11].

Within Panama, the Caribbean province of Colón bears a disproportionate burden of CMD [12]. Colón reports the highest national mortality rate from DM2 (35.8 per 100,000 in 2001–2011) [13], ischemic heart disease and stroke (41 and 35 per 100,000 in 2016, respectively) [14], and the second highest prevalence rate of obesity (26.2% in 2008) [7]. These figures underscore the urgent need to understand and address CMD in this region, particularly among vulnerable populations.

Concurrently, Panama faces a significant HIV epidemic, with a national prevalence rate of 1.1% among adults—almost twice the 0.6% estimate for the Latin American region [15]. Panama’s high prevalence rate of HIV infection has been reported in association with increased connection and access to sexual networks, condomless sexual encounters, and frequent, casual sexual encounters with multiple, new partners [16]. In 2023, HIV-related deaths accounted for 1.4% of all deaths in Panama [11]. Colón is also disproportionately affected, with the highest cumulative HIV prevalence rate in the country (580.6 per 100,000 in 2001–2020), and alarmingly higher rates among key populations, such as men who have sex with men, transgender women, and female sex workers [17,18,19]. Despite efforts to meet UNAIDS’ 95-95-95 targets by 2030, Panama is still falling short, with a 71% coverage with antiretroviral therapy (ART) estimated for 2025 [15].

As ART has extended the life expectancy of adults living with HIV (ALWH), the epidemiologic landscape has shifted. Noncommunicable diseases, particularly CMD, have emerged as leading health concerns in this population [20]. Infection with HIV induces chronic, low-grade systemic inflammation, which, along with ART-related metabolic effects, contributes to increased risk for MetS and CMD [21,22]. Elevated high-sensitivity C-reactive protein (hsCRP), an inflammatory biomarker, is commonly observed among ALWH and has been associated with MetS and its five individual components [23,24].

Beyond biological mechanisms, psychosocial stressors also play a critical role in the epidemiologic transition from HIV infection to CMD. The Minority Stress Theory posits that chronic exposure to stigma, discrimination, and marginalization contribute to adverse health outcomes among minoritized groups [25,26]. In Panama, ALWH—particularly men who have sex with men and transgender individuals—face significant barriers to healthcare access due to stigma and discrimination [18,19,27]. These bio-behavioral stressors may exacerbate inflammation and metabolic dysregulation, compounding the risk of CMD in this population [28,29,30].

Despite the overlapping epidemics of HIV and CMD, there is a critical gap in the literature regarding the prevalence and determinants of MetS among ALWH in Latin America, and Panama in particular. Two recent meta-analyses have estimated the pooled prevalence of MetS among ALWH. One study [31] conducted in 2020, with over one-third of its included data from 7 Latin American countries, estimated a 20.6% global MetS prevalence. Another study [32] conducted in 2024 found a global MetS prevalence estimate of 25.3%, with a higher pooled estimate (30.4%) among the 21 studies from the Americas. Recent studies found similar MetS prevalence estimates among ALWH in Guatemala (31.5% in 2022) [33] and Mexico (26.5% in 2025) [34]. The paucity of local data hampers the development of targeted interventions and clinical guidelines to address the unique CMD needs of ALWH in Panama.

To address this gap, the Colón C3 Study (*Salud Cardiometabólica en la Clínica de Colón*) was conducted at the ART Clinic in Colón City, the only ART clinic in the province serving an active patient population of 844 ALWH as of 2024. The Colón C3 Study aimed to estimate the prevalence and 12-month trajectory of MetS and its five criteria among ALWH in Colón, with the long-term goal to establish a baseline that will inform the development of culturally appropriate, evidence-based interventions and clinical guidelines that improve cardiometabolic health outcomes for ALWH in Panama, scalable to Latin America. This manuscript presents the baseline findings, focusing on the sociodemographic, anthropometric, and cardiometabolic characteristics of the study participants, and estimating the overall baseline prevalence of MetS and its diagnostic criteria among ALWH by sex assigned at birth in Colón.

## 2. Materials and Methods

### 2.1. Study Design and Setting

The Colón C3 Study is a single-site, observational cohort conducted at the ART Clinic in Colón, Panama (Supplementary Figure S1), to assess the sociodemographic, anthropometric, and cardiometabolic status of ALWH. Data were collected at baseline and two follow-up visits spaced 6 ± 1 months apart. This manuscript reports the baseline findings at enrollment (Visit 1). The full, detailed Colón C3 Study procedures are presented in Appendix A.

### 2.2. Ethical Oversight

This study was registered at the *Registro y Seguimiento de Investigación para la Salud* of Panama’s Ministry of Health (Registry No. 2722, dated 19 December 2019), approved and overseen by *Comité de Bioética de Investigación* at Pacífica Salud (Protocol No. 117, dated 18 March 2024), by the University of Central Florida’s Institutional Review Board (STUDY00006622, dated 25 April 2024).

### 2.3. Study Participants and Sample Size

Adults (≥18 years) with confirmed HIV diagnosis receiving care at the ART Clinic and residing in Colón province were eligible. Written informed consent was obtained from all participants. No quotas were applied for sex, age, or other sociodemographic characteristics. The target sample was 614 participants to account for a 15% attrition at every study visit and to ensure 80% power to detect a one-way 5% difference in MetS prevalence compared to the 30.4% most recent regional benchmark [32]. Of note, countries in this regional benchmark estimate did not include Panama.

### 2.4. Data Sources and Procedures

Participants completed a computer-assisted self-interview (CASI) in Spanish, capturing demographic data and social determinants of health (SDoH) using 13 validated instruments, detailed in Supplementary Table S1. Body measurements included height (cm), weight (kg), waist circumference (cm), and blood pressure (mmHg). Body composition was assessed using InBody 270 (InBody Co., Seoul, Republic of Korea), a direct segmental multi-frequency bioelectrical impedance analyzer (DSM-BIA) that measures impedance at 20 and 100 kHz across five body segments using an 8-point tactile electrode system [35]. Parameters assessed in the DSM-BIA (in kg) included body fat mass, fat-free mass, total body water, protein, minerals, and skeletal muscle mass. Body mass index (BMI, in kg/m^2^) was estimated from weight and height. Fasting blood samples were collected at every visit for glucose (mg/dL), triglycerides (mg/dL), and cholesterol (total, HDL, LDL, and VLDL, all in mg/dL). During the initial visit, HbA1c (%), hsCRP (mg/L), CD4+ count (cells/mL), and viral load (copies/mL) were also measured. The detailed list of anthropometric and laboratory variables is provided in Supplementary Table S2.

### 2.5. Outcomes and Statistical Analyses

*Study variables.* Metabolic syndrome (MetS) was defined using the five National Cholesterol Education Program Adult Treatment Program, 3rd edition, revised (NCEP-R ATP-III) criteria [36,37,38]: abdominal obesity, hypertension, hypertriglyceridemia, low HDL cholesterol, and hyperglycemia. Prevalence rates of MetS and its five criteria, along with their respective binomial 95% confidence intervals (95% CIs) were estimated and reported as percentages, as is the case for very frequent conditions [39]. Additional, related, proxy variables are also reported for each diagnostic criteria, even though these are not NCEP-R ATP-III criteria for MetS.

*Data management.* All the databases from the various sources and study visits were downloaded and merged by participant code, cleaned, and analyzed in SPSS v. 29.0 (The IBM Corporation; Armonk, NY, USA). Descriptive statistics involved summarization of continuous variables through means and standard deviations (SDs) and of categorical variables through frequencies and percentages. Comparisons by natal sex were conducted using independent samples *t* tests for continuous variables and independence chi-square (χ^2^) tests for categorical variables. Significance was set at the standard α ≤ 0.05. Given the cross-sectional design of this baseline report (Aim 1, Supplementary Figure S2), all analyses were intended to describe prevalence patterns and associations; no causal inference or mediation testing was conducted at this stage.

*Missing data.* CASI Instruments were completed by more than 95% of participants at the baseline visit, with missing data of fewer than 5% for each questionnaire and were assumed to be missing at random and treated via pair-wise deletions. The same rationale was applied to laboratory and anthropometric data, which had high completion rates (≥81% and ≥98%, respectively), with missing data of fewer than 5% for each individual variable.

## 3. Results

After 35 weeks of active recruitment, 734 patients of the ART Clinic were approached and invited to participate, representing an 87.0% outreach effort. Among the participants invited, 666 consented for study participation. Upon review of inclusion criteria, 7 consenting participants were excluded from the study: 3 did not have an HIV diagnosis, 3 were not residents of Colón, and 1 was inadvertently re-enrolled at Visit 1 upon their return for Visit 2. This participant’s re-enrollment code was voided and their data were transferred to the Visit 2 database under the original participant’s code. Thus, the baseline analytic sample was 659 participants (78.1% of the estimated 844 active patients in the ART Clinic), with an average recruitment rate of 19.0 participants/week. Results are presented as descriptive and sex-stratified associations, without assumptions of directionality. Table 1 summarizes the cascade of events from patient recruitment to the baseline and longitudinal analytic samples.

### 3.1. Demographic Characteristics

Table 2 summarizes the demographic characteristics of the analytic sample by natal sex. The analytic sample was demographically diverse, with a mean age of 43.85 (range 18–79) years, and an age distribution that approximated normality (skewness = 0.203; kurtosis = −0.544). Women had a longer duration of care at the ART Clinic and more minors in their households than men (*p* < 0.001 for both). Differences by natal sex were evident across civil status and socioeconomic indicators: men were more often single without a stable partner and more likely to be employed, have social security benefits, and private insurance, whereas women were more frequently widowed, reported lower incomes, and were more likely to indicate uncertainty about income (all *p* < 0.05). Racial identity also varied, with women more often identifying as Black/Afro-Caribbean. Housing patterns differed: women more frequently owned their homes outright, while men more often rented or lived with relatives (all *p* < 0.05).

### 3.2. Social Determinants of Health

Table 3 presents a summary of SDoH by natal sex in the baseline analytic sample. Women reported greater social cohesion and higher loneliness, while men experienced more everyday discrimination, particularly related to sexual orientation, race, and gender. Men also perceived greater neighborhood disorder (physical and social) and reported higher lifetime and recent substance use (alcohol, tobacco, marijuana, cocaine). In contrast, women had higher food insecurity and lower ART adherence self-efficacy, especially on the perseverance subscale.

### 3.3. Laboratory Measurements and HIV Treatment

Baseline laboratory data were available for over 82% of participants and are summarized in Table 4. Women showed higher abdominal circumference, BMI, and body fat mass, while men had greater fat-free and skeletal muscle mass, as well as higher VLDL and triglyceride levels. Women also had higher CD4+ counts, whereas hsCRP levels were similar across sexes. Nearly 90% of participants reported using single-tablet ART regimens, primarily Acriptega and Biktarvy (Table 5).

### 3.4. Prevalence of Metabolic Syndrome

The overall unadjusted baseline prevalence of MetS was 38.6% (95% CI: 34.5%, 42.9%; Table 6). The most common criteria were hypoalphalipoproteinemia and hypertension, followed by abdominal obesity, hyperglycemia, and hypertriglyceridemia. Additional markers included BMI ≥ 30 kg/m^2^ (29.3%) and HbA1c ≥ 6.5% (6.4%). Women had a markedly higher MetS prevalence than men (46.9% vs. 27.6, *p* < 0.001) and were more likely to meet multiple criteria. By component, women showed greater hypoalphalipoproteinemia and abdominal obesity (*p* < 0.001 for both), as well as higher rates of hypertension diagnosis and obesity (*p* < 0.05). Hyperglycemia and hypertriglyceridemia did not differ significantly by sex. These findings underscore a pronounced cardiometabolic risk among women living with HIV.

## 4. Discussion

The Colón C3 Study was designed to address a critical gap on the epidemiologic transition that ALWH experience in Latin America in general, and in Panama in particular, where CMD and MetS were hypothesized to be more prevalent than in the general population. Thus, this study provides a cross-sectional characterization of MetS, inflammatory markers, and social SDoH among ALWH in Colón—a province disproportionately affected by both HIV and CMD. Despite the growing burden of noncommunicable diseases among ALWH, few studies have systematically and simultaneously examined the prevalence and the SDoH and laboratory markers of MetS among ALWH. By focusing on a diverse cohort of ALWH in Colón—a province disproportionately affected by both HIV and CMD—this study provides essential data to advance the state of knowledge and to inform clinical practice and public health interventions. While the findings reveal a substantial burden of MetS and marked differences by natal sex, the analyses are descriptive and associational in nature. Accordingly, observed relationships between social, behavioral, inflammatory, and cardiometabolic factors should be interpreted as contextual and hypothesis-generating rather than causal.

### 4.1. Sociodemographic Profile

The sociodemographic profile of the study cohort supports the representativeness of the sample, recruiting 78.1% of the active patient population at the ART Clinic in Colón. The cohort was socio-demographically diverse, with a majority identifying as Black/Afro-Caribbean. Gender identity largely aligned with natal sex, and sexual orientation varied, with 13.9% identifying as gay/lesbian and 5.4% as bisexual. Compared to the national average [40], educational attainment was high, with 43.2% having completed high school and 11.9% holding a bachelor’s degree or higher. However, employment and income indicators revealed substantial socioeconomic disparities. These structural inequities mirror the broader general population of Colón [41], enhancing the external validity of the findings. Inequities like these have been posited as contributors to the development of CMD [14] and must be considered in the design of public health interventions for ALWH.

The health profile of ALWH in the ART Clinic further helps contextualize the CMD findings. The mean age at HIV diagnosis was 31.7 years, and participants had been patients at the ART Clinic for an average of 8.9 years. Most participants were receiving single-tablet ART regimens, provided by formularies in either the Ministry of Health (Acriptega) or the Social Security Fund (Biktarvy). The HIV-Treatment ASES scores were slightly lower among women, suggesting potential natal sex–based barriers to optimal ART adherence.

### 4.2. Social Determinants of Health

Notably, SDoH also emerged as salient contextual factors associated with CMD in this cohort. Higher levels of food insecurity, housing instability, housing quality problems, perceived discrimination, and neighborhood disorder were observed alongside elevated CMD burden. These indicators of economic vulnerability are known to correlate with poor dietary quality, reduced physical activity, and limited healthcare access—all of which contribute to CMD [42]. Women reported significantly higher levels of social cohesion and loneliness, while men experienced significantly higher perceived discrimination, particularly related to sexual orientation, race, and gender. Perceived neighborhood disorder was significantly higher among men. In Colón, where Afro-Caribbean and LGBTQ+ communities are disproportionately affected by HIV, these stressors may amplify CMD risk through known biobehavioral pathways that generally align with the Minority Stress Theory—which posits that chronic exposure to stigma and marginalization contributes to adverse health outcomes [25,26,27,28]. In the context of HIV, these and other minority stressors (e.g., majority Afro-Caribbean race, majority women, closeted sexual orientation, and poverty despite high educational attainment, among others) coexist as demonstrated in the current study and may exacerbate inflammation and metabolic dysregulation, compounding CMD risk, as demonstrated by others [28,43,44]. Additionally, substance use patterns revealed high lifetime and recent use of alcohol, tobacco, and marijuana, particularly among men. While the prevalence of other substances use was low, the cumulative burden of substance use may interact with ART adherence and metabolic outcomes. The integration of SDoH assessments into HIV primary care may facilitate more holistic interventions that address upstream determinants of health.

### 4.3. Laboratory Findings

Laboratory findings provide further insight into the biological mechanisms underlying MetS. The mean fasting blood glucose level (109 mg/dL) and HbA1c (5.8%) point to early metabolic dysregulation with insulin resistance, compatible with reports from ALWH in Puerto Rico [45], but lower than those reported for the general population in Panama [12,13,14]. Lipid profiles revealed hypoalphalipoproteinemia (mean HDL cholesterol 47 mg/dL) and elevated triglycerides (116 mg/dL), consistent with prior studies linking HIV infection and ART exposure to dyslipidemia [46,47,48,49]. ART regimens, particularly those containing protease inhibitors and certain integrase strand transfer inhibitors (INSTIs), have been associated with lipid abnormalities [50] compatible with those observed in this study. Although most participants were on single-tablet regimens—such as the more metabolically favorable Acriptega and Biktarvy—the persistence of dyslipidemia suggests that ART alone does not fully mitigate CMD risk. As it has been shown, even newer ART regimens can contribute to lipid abnormalities and insulin resistance, particularly in the context of chronic inflammation and lifestyle factors [51], underscoring the need for routine lipid monitoring and potential lifestyle and pharmacologic interventions. As a marker of systemic inflammation, hsCRP averaged 7.2 mg/L, with substantial variability, suggesting a persistent inflammatory milieu despite high rates of viral suppression. Elevated hsCRP values have been linked to MetS and its individual criteria among ALWH, supporting the relevance of chronic inflammation in the pathogenesis of CMD among ALWH [22,24,49]. However, in this baseline analysis, hsCRP should be interpreted as an associated biomarker reflecting co-occurring inflammation rather than evidence of a causal or mediating inflammatory pathway, as temporality and mechanistic relationships cannot be established cross-sectionally.

### 4.4. Metabolic Syndrome and Individual Criteria

The Colón C3 Study reveals a substantial burden of MetS among ALWH in Panama, with a prevalence of 38.6%, surpassing recent estimates for the Americas (30.4%) [32], Guatemala (31.5%) [33], and Mexico (26.5%) [34]. This elevated rate highlights the epidemiologic transition in Panama, where noncommunicable diseases increasingly dominate morbidity and mortality patterns. The CMD risk was pronounced: 21.2% met three criteria, and nearly one-fifth met four or all five for MetS, signaling advanced CMD risk clustering. Compared to ART-naïve populations—where MetS prevalence tends to be lower due to younger age and shorter HIV/ART exposure—the Colón C3 Study cohort reflects a more chronic CMD profile, consistent with the cohort’s mean 8.93 years living with HIV. These findings draw attention to the need for integrated HIV and CMD prevention and care models tailored to long-term survivors.

Among individual criteria, hypoalphalipoproteinemia (59.6%) and hypertension (52.6%) were most prevalent, followed by abdominal obesity (45.2%), hyperglycemia (33.5%), and hypertriglyceridemia (22.5%), aligning with global evidence linking HIV and ART to dyslipidemia and blood pressure dysregulation [46,47,48]. Elevated hsCRP further supports chronic inflammation as a key driver of MetS pathogenesis among ALWH, consistent with prior studies showing persistent inflammatory activation despite virologic suppression [22,24,52]. Abdominal obesity exceeded national estimates for the general population (45.2% vs. 3.9%) [10], compounding CMD risk. Differences by natal sex were striking: women exhibited significantly higher body fat and BMI, consistent with global trends in obesity among women—both in the general population [53] and among women living with HIV worldwide [2] and in Panama [54]. These disparities may reflect natal-sex differences in diet, physical activity, and access to preventive care, reinforcing calls for targeted interventions in resource-limited settings like Colón [14]. Hyperglycemia and hypertriglyceridemia prevalence mirror Panama’s broader shift toward noncommunicable disease dominance [55], with fasting glucose and HbA1c levels suggesting early metabolic dysregulation potentially exacerbated by chronic inflammation—key contributors to CMD pathogenesis among ALWH [22,24,52]. While the cross-sectional nature of these analyses limits causal inference, the observed patterns generate testable hypotheses regarding biobehavioral mechanisms linking HIV, chronic stress, inflammation, and metabolic health. Ongoing longitudinal analyses are warranted to disentangle temporality and assess the role of these factors on CMD trajectories.

### 4.5. Differences by Natal Sex

Marked differences by natal sex were observed across MetS outcomes and social context among ALWH. Women exhibited a substantially higher prevalence of metabolic syndrome and were more likely to meet multiple diagnostic criteria, largely driven by greater abdominal adiposity, higher body fat mass, and obesity. These findings are consistent with prior evidence indicating natal sex–specific patterns of fat distribution and metabolic vulnerability among women living with HIV, potentially accentuated over prolonged ART exposure [2,20,53]. Although inflammatory markers such as hsCRP were elevated overall, levels were comparable by natal sex, suggesting that differential cardiometabolic risk may not be explained by inflammation alone [22,24]—gendered social and structural factors likely further contextualize these disparities. Women reported significantly higher food insecurity, lower income stability, and reduced ART adherence self-efficacy, all of which have been linked to cardiometabolic risk [14,42]. Collectively, these findings indicate that natal sex–based disparities in cardiometabolic health among ALWH reflect the intersection of biological susceptibility and social vulnerability. While descriptive, these observations generate hypotheses that will be examined longitudinally.

### 4.6. Limitations

This study is not void of limitations that should be acknowledged. First, while the cross-sectional design is valuable for estimating prevalence rates, it precluded causal inference [56], even though the associations between CMD markers and SDoH are self-evident and supported in the literature. Accordingly, any interpretation of potential pathways linking SDoH, inflammation, ART exposure, and MetS should be considered hypothesis-generating and not evidence of causation or mediation. Thus, longitudinal analyses are needed to establish temporality and directionality. Second, although the sample size was robust and representative of the ART Clinic patient population, generalizability to other regions of Panama or Latin America may be limited. The unique sociodemographic and epidemiologic context of Colón necessitates caution in extrapolating findings. Third, self-reported data from the CASI may be subject to social desirability and recall biases [57,58], particularly for sensitive topics where nonresponses were high (e.g., income, sexual orientation, and illegal substance use). While robust, validated instruments were used in the study [59], said biases may affect the accuracy and consistency of responses recorded. Fourth, laboratory data completion rates varied, with some biomarkers missing for up to 18% of participants. Although data were treated as missing at random and addressed via pairwise deletion, this may introduce bias [57,58], especially in the prevalence estimates for laboratory-driven MetS criteria (i.e., HDL cholesterol and triglycerides). While the potential biases introduced by pairwise deletion of missing data are minor in the descriptive outcomes for this study [57,58], analysis of data missingness patterns, selective nonresponse, and sensitivity analyses are warranted for the ongoing longitudinal analyses and effect size estimation. Fifth, the study did not include a control group of HIV-negative individuals, limiting our ability to compare CMD prevalence by HIV status. Despite our best efforts to identify benchmark MetS prevalence data from Panama and Colón, we could not identify peer-reviewed or agency publications with recent, comparable data. Future studies should consider either matched controls to better isolate the impact of HIV and ART on CMD or repeat similar methodologies across the general population, controlling for HIV serostatus. Sixth, the study used NCEP-R ATP-III cut-offs for waist circumference, which may misclassify cardiometabolic risk in Afro-descendant populations [60], particularly among women [61], as demonstrated by multiple HIV-specific [62,63] and Latin American validation studies [64,65]. In the absence of Panama-specific thresholds, NCEP-R ATP-III criteria were applied to ensure comparability with the existing HIV literature, underscoring the urgent need for locally derived, population-specific validation. Finally, while hsCRP was measured, other inflammatory markers—such as interleukin 6 (IL 6) or tumor necrosis factor alpha (TNF α)—were not included. While it can be cost-prohibitive, a more comprehensive inflammatory and metabolic laboratory profile would enhance understanding of the biological mechanisms linking HIV to CMD.

### 4.7. Strengths

Despite these limitations, the Colón C3 Study also presents several notable strengths that enhance its scientific rigor and relevance. First, the study achieved a high recruitment rate (78.1% of the clinic’s active patient population) which, along the design without quotas—by natal sex, age, time living with HIV, gender identity, or sexual orientation—ensured inclusiveness and representativeness of ALWH in Colón. Second, to the best of our knowledge, this is one of the largest and most comprehensive assessments of CMD among ALWH in Panama and Latin America, with high return rates (attrition < 13% in every visit) and high completion rates for survey, laboratory, and anthropometric datasets. Third, the use of validated instruments from the *All of Us* Research Program [59] allowed for robust assessment of SDoH, a critical yet often underexplored domain in HIV research. Fourth, the implementation of DSM-BIA for body composition [35] and certified laboratory testing provided objective, high-quality data on CMD biomarkers. While dual X-ray absorptiometry (DXA) is considered the gold standard for body composition analyses [66], DSM-BIA shows high reliability and affordability for adults in the general population [67] and in ALWH [68]. Finally, while not reported in this manuscript, the longitudinal structure of the study, with repeated measures planned over 12 months, positions the Colón C3 Study to generate temporally rich data that can inform causal models and trends over time. Moreover, triangulation with qualitative data from focus groups with participants and personnel will support the development of intervention strategies tailored to the evolving needs of ALWH in Panama.

## 5. Conclusions

The Colón C3 Study provides critical baseline data on the cardiometabolic health of ALWH in Panama, revealing a high prevalence of MetS (38.6%) and its five criteria, with corresponding biomarkers and SDoH. These findings highlight the need for integrated care models that address both HIV and CMD, incorporating routine screening, lifestyle interventions, and pharmacologic management. The intersection of biological, behavioral, and SDoH underscores the complexity of CMD in ALWH. Beyond clinical biomarkers, addressing associated stigma, discrimination, and economic vulnerability is essential to improving health outcomes. Policymakers and clinicians must prioritize culturally tailored interventions that reflect the lived experiences of ALWH in Colón.

Future research should leverage the longitudinal design of the Colón C3 Study to examine trajectories of CMD and evaluate the moderating role of chronic inflammation. By bridging gaps in the literature and informing clinical practice, this study contributes to the broader goal of health equity for ALWH in Panama and Latin America.

## Figures and Tables

**Table 1:** Cascade of events from patient recruitment until completion of the baseline and longitudinal analytic samples.

Study Events	*N*	Percentage of		
		ART Clinic Active Patients (*N* = 844)	Invited Patients (*N* = 734)	Baseline AS (*N* = 659)
Estimated ART Clinic active patients	844	100%	—	—
Patients approached/invited	734	87.0%	100%	—
Consenting participants	666	78.9%	90.7%	—
Participants not meeting inclusion criteria	7	0.8%	1.0%	—
Participants included in Visit 1 **(baseline AS)**	659	78.1%	89.8%	100%
Deaths reported between Visits 1–2	7	0.8%	1.0%	1.1%
Losses to follow-up at Visit 2	84	10.0%	11.4%	12.7% ^a^
Participants returning to Visit 2	562	66.6%	76.6%	85.3%
Deaths reported between Visits 2–3	5	0.6%	0.7%	0.9% ^b^
Losses to follow-up at Visit 3	62	7.3%	8.4%	11.0% ^a,b^
Participants returning to Visit 3 **(longitudinal AS)**	495	58.6%	67.4%	88.1% ^b^

Abbreviations: ART, antiretroviral therapy; AS, analytic sample. ^a^ Attrition rate between visits. ^b^ Calculated with 562 participants returning to Visit 2 as the denominator.

**Table 2:** Sociodemographic characteristics of study participants by natal sex.

Continuous Variables	Natal Sex						*p* Value ^a^
	Male (*N* = 296)		Female (*N* = 363)		Total (*N* = 659)		
	*n*	%	*n*	%	*n*	%	
Age, y	43.58	13.29	44.07	11.79	43.85	12.48	0.660
Median, IQR	44	33, 54	44	33, 52	44	34, 53	—
Adults in the household (≥18 y)	2.74	1.98	2.80	1.78	2.77	1.87	0.683
Minors in the household (<18 y)	1.02	1.52	1.72	1.91	1.40	1.78	<0.001
Age when diagnosed with HIV, y	32.49	12.26	31.05	11.77	31.69	12.01	0.126
Time as patient in the ART Clinic, y	8.06	5.86	9.63	5.93	8.93	5.94	<0.001
**Categorical Variables**	**Natal Sex**						***p*** **Value ^b^**
	**Male (*N* = 296)**		**Female (*N* = 363)**		**Total (*N* = 659)**		
	***n***	**%**	***n***	**%**	***n***	**%**	
Gender identity							
Male	287	97.62%	3	0.83%	290	44.14%	<0.001
Female	6	2.04%	360	99.17%	366	55.71%	<0.001
Nonbinary	1	0.34%	—	—	1	0.15%	NE
Transexual identity	6	2.04%	3	0.83%	9	1.37%	0.193
Sexual orientation							
Heterosexual	141	56.63%	259	88.70%	400	73.94%	<0.001
Gay or lesbian	71	28.51%	4	1.37%	75	13.86%	<0.001
Bisexual	26	10.44%	3	1.03%	29	5.36%	<0.001
Asexual	3	1.20%	13	4.45%	16	2.96%	0.016
Other	8	3.21%	13	4.45%	21	3.88%	0.418
Prefers not to answer	27	9.38%	33	9.51%	60	9.45%	0.955
Does not know how to answer	12	4.17%	22	6.34%	34	5.35%	0.224
Race ^c^							
Black/Afro-Caribbean	136	45.95%	203	55.92%	339	51.44%	0.012
Mestizo/Mestiza	93	31.42%	96	26.45%	189	28.68%	0.167
White	28	9.46%	38	10.47%	66	10.02%	0.672
Native Panamanian	22	7.43%	16	4.41%	38	5.77%	0.104
Asian	6	2.03%	2	0.55%	8	1.21%	0.092
More than one race	5	1.69%	9	2.48%	14	2.12%	0.489
Does not know how to answer	4	1.35%	6	1.65%	10	1.52%	0.757
Prefers not to answer	8	2.70%	8	2.20%	16	2.43%	0.683
Civil status							
Single, without a stable partner	188	63.73%	171	47.11%	359	54.56%	<0.001
Single, with a stable partner	46	15.59%	78	21.49%	124	18.84%	0.057
Common law for ≥5 years	21	7.12%	32	8.82%	53	8.05%	0.431
Married	29	9.83%	51	14.05%	80	12.16%	0.103
Divorced	5	1.69%	4	1.10%	9	1.37%	0.523
Widow/Widower	6	2.03%	27	7.44%	33	5.02%	0.002
Pregnant	—	—	11/350	3.00%	—	—	NE
Currently working	144	49.48%	83	22.87%	227	34.71%	<0.001
Has social security benefits	114	38.91%	104	28.65%	218	33.23%	0.006
Has private health insurance	19	6.57%	10	2.79%	29	4.48%	0.022
Monthly income, PAB ^d^							
<250	62	22.22%	110	32.26%	172	27.74%	0.004
250–500	51	18.28%	32	9.38%	83	13.39%	0.001
501–750	48	17.20%	15	4.40%	63	10.16%	<0.001
751–1000	16	5.73%	13	3.81%	29	4.68%	0.253
>1000	12	4.30%	5	1.47%	17	2.74%	0.030
Prefers not to answer	64	22.94%	98	28.74%	162	26.13%	0.096
Does not know how to answer	26	9.32%	68	19.94%	94	15.16%	<0.001
Highest school level completed							
Incomplete elementary (<6 grades)	13	4.39%	31	8.56%	44	6.69%	0.035
Elementary (6 grades)	40	13.51%	56	15.47%	96	14.59%	0.484
General basic education (9 grades)	36	12.16%	51	14.09%	87	13.22%	0.473
High school (12 grades)	127	42.91%	157	43.37%	284	43.16%	0.907
Associate’s degree	23	7.77%	24	6.63%	47	7.14%	0.578
Bachelor’s degree	45	15.20%	33	9.12%	78	11.85%	0.018
Graduate certificate	2	0.68%	1	0.28%	3	0.46%	0.459
Master’s degree	3	1.01%	—	—	3	0.46%	NE
Prefers not to answer	4	1.35%	6	1.66%	10	1.52%	0.749
Does not know how to answer	3	1.01%	3	0.83%	6	0.91%	0.812
Housing							
Owns, fully paid	107	36.27%	171	47.50%	278	42.44%	0.004
Owns, paying mortgage	33	11.19%	47	13.06%	80	12.21%	0.472
Informal without property rights	14	4.75%	28	7.78%	42	6.41%	0.118
Rental/lease	53	17.97%	30	8.33%	83	12.67%	<0.001
Lives with relatives	73	24.75%	61	16.94%	134	20.46%	0.015
Prefers not to answer	8	2.71%	8	2.22%	16	2.44%	0.690
Does not know how to answer	7	2.37%	15	4.17%	22	3.36%	0.207

Abbreviations: ART, antiretroviral therapy; HIV, human immunodeficiency virus; IQR, interquartile range; NE, not estimable; PAB, Panamanian Balboa; SD, standard deviation; USD, United States Dollar; y, years. ^a^ *t* test with 657 df for the comparison of means (SD) by natal sex. ^b^ Chi-squared test with 1 df for the comparison of proportions by natal sex. ^c^ Percentages may not add up to 100%, as participants could select more than one answer. ^d^ One 2024 PAB is equivalent to one 2024 USD.

**Table 3:** Social determinants of health measures of study participants by natal sex.

Social Determinants of Health Measures	Natal Sex, Mean (SD)			*p* Value ^a^
	Male (*N* = 296)	Female (*N* = 363)	Total (*N* = 659)	
**Social and Community Context**				
Social Cohesion Neighborhood Scale	2.66 (0.95)	2.88 (1.00)	2.78 (0.99)	0.004
MOS Social Support Survey	3.87 (1.03)	3.95 (1.13)	3.91 (1.09)	0.347
UCLA Loneliness Scale ^b^	2.34 (0.52)	2.45 (0.53)	2.40 (0.53)	0.008
The Everyday Discrimination Scale + Attribution	0.91 (1.15)	0.65 (0.94)	0.77 (1.05)	0.002
Education or income level	25 (8.5%)	34 (9.5%)	59 (9.1%)	0.660 ^c^
Sexual orientation	34 (11.6%)	9 (2.5%)	43 (6.6%)	<0.001 ^c^
Ancestry or national origins	24 (8.2%)	16 (4.5%)	40 (6.2%)	0.054 ^c^
Some other aspect of physical appearance	17 (5.8%)	21 (5.9%)	38 (5.8%)	0.957 ^c^
Age	14 (4.8%)	18 (5.0%)	32 (4.9%)	0.907 ^c^
Race	20 (6.8%)	9 (2.5%)	29 (4.5%)	0.009 ^c^
Gender	22 (7.5%)	5 (1.4%)	27 (4.2%)	<0.001 ^c^
Weight	9 (3.1%)	14 (3.9%)	23 (3.5%)	0.585 ^c^
Religion	8 (2.7%)	14 (3.9%)	22 (3.4%)	0.401 ^c^
Height	7 (2.4%)	1 (0.3%)	8 (1.2%)	0.018 ^c^
Other (specify)	19 (6.5%)	26 (7.3%)	45 (6.9%)	0.692 ^c^
HIV status	3 (15.8%)	7 (26.9%)	10 (22.2%)	<0.001 ^c^
Cohen’s Perceived Stress Scale	26.96 (6.52)	27.13 (5.68)	27.05 (6.06)	0.721
**Neighborhood and Built-in Environment**				
Ross–Mirowsky Perceived Neighborhood Disorder Scale ^b^				
Physical Disorder Subscale, 6 items	3.63 (0.74)	3.45 (0.77)	3.54 (0.76)	0.003
Social Disorder Subscale, 7 items	3.35 (0.80)	3.20 (0.83)	3.27 (0.82)	<0.001
Physical Activity Neighborhood Environment Scale (PANES)				
Neighborhood walkability subscale, ^b^ 5 core items	2.23 (0.81)	2.30 (0.83)	2.27 (0.82)	0.277
Neighborhood crime subscale, ^b^ 2 items	2.96 (1.36)	3.17 (1.38)	3.08 (1.37)	0.051
Neighborhood residential density, 1 item				
Single family	167 (60.5%)	188 (58.4%)	355 (59.4%)	0.590 ^c^
All other	109 (39.5%)	134 (41.6%)	243 (40.6%)	0.590 ^c^
**Economic Stability**				
Children’s HealthWatch Hunger vital sign	70 (24.1%)	123 (34.8%)	193 (30.0%)	0.003 ^c^
Housing Stability Indicator	0.42 (1.32)	0.45 (1.26)	0.43 (1.29)	0.766
Housing Quality Problems	97 (38.2%)	138 (42.2%)	235 (40.4%)	0.304 ^c^
**Health and Healthcare**				
Discrimination in healthcare settings, 7-item	1.45 (0.59)	1.44 (0.55)	1.45 (0.57)	0.822
HIV-Treatment Adherence Self-Efficacy Scale (ASES)	9.49 (1.39)	9.23 (1.73)	9.34 (1.59)	0.037
Integration subscale, 9 items	9.48 (1.40)	9.25 (1.72)	9.35 (1.59)	0.064
Perseverance subscale, 3 items	9.53 (1.37)	9.16 (1.92)	9.32 (1.70)	0.006
Alcohol, Smoking and Substance Involvement Screening Test-Frequency and Concern (ASSIST-FC), Substance-specific Involvement (SSI)				
Alcoholic beverage use				
Lifetime	221 (79.8%)	216 (62.8%)	437 (70.4%)	<0.001 ^c^
Past 12 mo	172 (77.8%)	139 (64.4%)	311 (71.1%)	<0.001 ^c^
Tobacco product use				
Lifetime	69 (25.3%)	19 (5.6%)	88 (14.3%)	<0.001 ^c^
Past 12 mo	51 (73.9%)	14 (73.7%)	65 (73.9%)	0.978 ^c^
Marijuana use				
Lifetime	60 (20.7%)	21 (5.9%)	81 (12.6%)	<0.001 ^c^
Past 12 mo	18 (30.0%)	5 (23.8%)	23 (28.4%)	0.590 ^c^
Cocaine use				
Lifetime	35 (12.1%)	13 (3.7%)	48 (7.5%)	<0.001 ^c^
Past 12 mo	7 (20.0%)	4 (30.8%)	11 (22.9%)	0.434 ^c^
All other drugs use ^d^				
Lifetime	7 (3.2%)	5 (2.3%)	12 (2.7%)	0.479 ^c^
Past 12 mo	4 (57.1%)	3 (60.0%)	7 (58.3%)	0.923 ^c^

Abbreviations: df, degrees of freedom; HIV, human immunodeficiency virus; mo, month; MOS, Medical Outcomes Study; UCLA, University of California-Los Angeles. ^a^ *t* test with 657 df for the comparison of means (SD) by natal sex, unless otherwise specified. ^b^ Modified to match 5-point Likert scales consistently. ^c^ Chi-squared test with 1 df for the comparison of proportions in male vs. female participants. ^d^ Includes: sedatives/sleeping pills, inhalants, methamphetamines, prescriptions stimulants, and hallucinogens.

**Table 4:** Anthropometric and laboratory measurements of study participants by natal sex.

Measurements	Natal Sex, Mean (SD)			*p* Value ^a^
	Male (*N* = 296)	Female (*N* = 363)	Total (*N* = 659)	
**Anthropometric**				
Blood pressure, mmHg				
Systolic	121.4 (19.2)	120.9 (20.0)	121.1 (19.6)	0.745
Diastolic	81.0 (11.3)	79.4 (11.8)	80.2 (11.6)	0.078
Abdominal circumference, cm	91.2 (14.9)	95.6 (17.1)	93.6 (16.3)	<0.001
Weight, kg	74.8 (16.1)	73.4 (20.4)	74.0 (18.6)	0.337
Height, cm	169.9 (9.8)	159.0 (10.4)	163.9 (11.5)	<0.001
BMI, kg/m^2^	26.0 (5.5)	29.2 (9.0)	27.8 (7.8)	<0.001
Body fat mass, kg	19.3 (10.2)	29.6 (13.9)	25 (13.4)	<0.001
Fat-free mass, kg	55.5 (10.0)	43.8 (9.4)	49.0 (11.3)	<0.001
Total body water, kg	40.8 (7.3)	32.2 (7.1)	36.0 (8.4)	<0.001
Protein, kg	11.0 (2.0)	8.6 (1.9)	9.7 (2.3)	<0.001
Minerals, kg	3.7 (0.7)	3.0 (0.7)	3.3 (0.8)	<0.001
Skeletal muscle mass, kg	31.2 (6.0)	23.9 (5.7)	27.2 (6.9)	<0.001
**Laboratory**				
Fasting blood glucose, mg/dL	108.1 (34.0)	109.0 (35.6)	108.6 (34.9)	0.742
HbA1c, %	5.7 (0.7)	6.0 (4.0)	5.8 (3.0)	0.203
Cholesterol, mg/dL				
Total	168.5 (39.6)	170.0 (37.2)	169.3 (38.3)	0.617
VLDL	25.1 (13.8)	22.0 (10.2)	23.4 (12.0)	0.001
LDL	98.4 (33.1)	104.0 (84.1)	101.5 (66.4)	0.281
HDL	45.8 (15.1)	47.7 (13.6)	46.8 (14.3)	0.090
Triglycerides, md/dL	122.8 (63.6)	111.0 (54.3)	116.3 (58.9)	0.010
hsCRP, mg/L	6.6 (6.0)	7.7 (8.3)	7.2 (7.4)	0.057
CD4+ cell count, cells/mm^3^	525.7 (272.6)	601.1 (340.3)	567.5 (313.9)	0.002
Viral load, ^b^ ×10^3^ copies/mm^3^	798.1 (12,466.7)	6.4 (48.1)	355.3 (8276.4)	0.227
Undetectable (<40 copies/mm^3^), *n* (%)	201 (77.3%)	273 (82.7%)	474 (80.3%)	0.088 ^c^

Abbreviations: BMI, body mass index; CD4+, cluster differentiation receptor type 4; HbA1c, hemoglobin A1c; HDL, high-density lipoprotein; hsCRP, high-sensitivity C-reactive protein; LDL, low-density lipoprotein; VLDL, very low–density lipoprotein. ^a^ *t* test with 657 df for the comparison of means (SD) by natal sex. ^b^ For calculation purposes, laboratory results reported as undetectable were scored as 0 and presented below for the lower detection limits in the laboratory (i.e., <20 and <40 copies/mm^3^). ^c^ Chi-squared test with 1 df for the comparison of proportions by natal sex.

**Table 5:** HIV ART reported by study participants by natal sex.

HIV ART ^a^	Natal Sex, *N* (%)						*p* Value ^b^
	Male (*N* = 296)		Female (*N* = 363)		Total (*N* = 659)		
	*n*	%	*n*	%	*n*	%	
**Combination, single-tablet regimens**							
Acriptega (dolutegravir 50 mg, lamivudine 300 mg, tenofovir 300 mg)	174	58.8%	225	62.0%	399	60.5%	0.404
Biktarvy (bictegravir 50 mg, emtricitabine 200 mg, tenofovir alafenamide 25 mg)	90	30.4%	84	23.1%	174	26.4%	0.035
**Combination, used with other ART**							
Combivir (lamivudine 150 mg, zidovudine 300 mg)	8	2.7%	16	4.4%	24	3.6%	0.247
Kaletra (lopinavir 200 mg, ritonavir 50 mg)	3	1.0%	14	3.9%	17	2.6%	0.020
Truvada (emtricitabine 200 mg, tenofovir disoproxil fumarate 300 mg)	3	1.0%	7	1.9%	10	1.5%	0.344
Prezcobix (darunavir 800 mg, cobicistat 150 mg)	2	0.7%	5	1.4%	7	1.1%	0.389
**Protease inhibitors**							
Prezista (darunavir 400, 600, or 800 mg)	4	1.4%	5	1.4%	9	1.4%	>0.999
**INSTIs**							
Tivicay (dolutegravir 25 or 50 mg)	3	1.0%	2	0.6%	5	0.8%	0.786

Abbreviations: ART, antiretroviral treatment; df, degrees of freedom; HIV, human immunodeficiency virus; INSTI, integrase strand transfer inhibitor. ^a^ Limited to the formulary available at the Colón C3 Study Site (ART Clinic) during the study. ^b^ Chi-squared test with 1 df for the comparison of proportions by natal sex.

**Table 6:** Unadjusted prevalence rate estimates of MetS, its five NCEP-R ATP-III diagnostic criteria, and associated proxies by natal sex.

MetS & CMD Markers ^a^	Unadjusted Prevalence Rate Estimates by Natal Sex						*p* Value ^a^
	Male		Female		Total		
	*n*/Valid *N* (%)	Binomial 95% CI	*n*/Valid *N* (%)	Binomial 95% CI	*n*/Valid *N* (%)	Binomial 95% CI	
**Metabolic syndrome**	**61/221 (27.6%)**	**22.0%, 33.8%**	**138/294 (46.9%)**	**41.3%, 52.7%**	**199/515 (38.6%)**	**34.5%, 42.9%**	**<0.001**
3 criteria	34/221 (15.4%)	11.1%, 20.6%	75/294 (25.5%)	20.8%, 30.7%	109/515 (21.2%)	17.8%, 24.8%	0.006
4 criteria	22/221 (10.0%)	6.5%, 14.4%	50/294 (17.0%)	13.0%, 21.6%	72/515 (14.0%)	11.2%, 17.2%	0.023
5 criteria	5/221 (2.3%)	0.9%, 5.9%	13/294 (4.4%)	2.5%, 7.2%	18/515 (3.5%)	2.2%, 5.4%	0.200
**Hypoalphalipoproteinemia**	**115/241 (47.7%)**	**41.5%, 54.0%**	**211/300 (70.3%)**	**65.0%, 75.3%**	**326/547 (59.6%)**	**55.4%, 63.7%**	**<0.001**
HDL cholesterol ^b^ ♂ < 40 mg/dL/♀ < 50 mg/dL	100/258 (38.8%)	33.0%, 44.8%	196/320 (61.3%)	55.8%, 66.5%	296/578 (51.2%)	47.1%, 55.3%	<0.001
Self-reported medication use for cholesterol	20/242 (8.3%)	5.3%, 12.2%	31/214 (14.5%)	10.3%, 19.7%	51/556 (9.2%)	7.0%, 11.8%	0.036
Self-reported hypercholesterolemia diagnosis ^c^	43/243 (17.7%)	13.3%, 22.9%	75/313 (24.0%)	19.5%, 28.9%	118/556 (21.2%)	18.0%, 24.8%	0.072
**Hypertension**	**130/256 (50.8%)**	**44.7%, 56.9%**	**177/328 (54.0%)**	**48.6%, 59.3%**	**307/584 (52.6%)**	**48.5%, 56.6%**	**0.443**
SBP ≥ 130 mmHg	93/294 (31.6%)	26.5%, 37.1%	103/363 (28.4%)	23.9%, 33.2%	196/657 (29.8%)	26.4%, 33.4%	0.244
DBP ≥ 85 mmHg	79/294 (26.9%)	22.0%, 32.1%	91/363 (25.1%)	20.8%, 29.7%	170/657 (25.9%)	22.6%, 29.3%	0.601
Self-reported hypertension diagnosis	68/242 (28.1%)	22.7%, 34.0%	118/314 (37.6%)	32.4%, 43.0%	186/556 (33.5%)	29.6%, 37.5%	0.019
Self-reported medication use for hypertension	41/242 (16.9%)	12.6%, 22.0%	68/314 (21.7%)	17.4%, 26.5%	109/556 (19.6%)	16.5%, 23.1%	0.141
**Abdominal obesity**	**64/292 (21.9%)**	**17.5%, 26.9%**	**230/359 (64.1%)**	**59.0%, 68.9%**	**294/651 (45.2%)**	**41.4%, 49.0%**	**<0.001**
Waist circumference ♂ > 102 cm/♀ > 88 cm	64/292 (21.9%)	17.5%, 26.9%	230/359 (64.1%)	59.0%, 68.9%	294/651 (45.2%)	41.4%, 49.0%	<0.001
Self-reported obesity diagnosis ^c^	77/243 (31.7%)	26.1%, 37.7%	152/314 (48.4%)	42.9%, 53.9%	229/557 (41.1%)	37.1%, 45.2%	<0.001
Self-reported medication use for obesity ^c^	5/242 (2.1%)	0.8%, 4.5%	9/214 (4.2%)	2.1%, 7.5%	14/556 (2.5%)	1.5%, 4.1%	0.196
BMI ^c^ > 30 kg/m^2^	53/292 (18.2%)	14.1%, 22.9%	138/359 (38.4%)	33.5%, 43.5%	191/651 (29.3%)	25.9%, 32.9%	<0.001
**Hyperglycemia**	**78/237 (32.9%)**	**27.2%, 39.1%**	**104/307 (33.9%)**	**28.8%, 39.3%**	**182/544 (33.5%)**	**29.6%, 37.5%**	**0.807**
Blood glucose ^b^ ≥ 110 mg/dL	67/264 (25.4%)	20.4%, 30.9%	88/331 (26.6%)	22.0%, 31.5%	155/595 (26.1%)	22.6%, 29.7%	0.741
Self-reported DM2 diagnosis	32/243 (13.2%)	9.4%, 17.8%	43/314 (13.7%)	10.2%, 17.8%	75/557 (13.5%)	10.8%, 16.5%	0.864
Self-reported medication use for blood sugar	18/242 (7.4%)	4.6%, 11.3%	17/314 (5.4%)	3.3%, 8.3%	35/556 (6.3%)	4.5%, 8.5%	0.335
HbA1c ^b,c^ ≥ 6.5%	15/263 (5.7%)	3.4%, 9.0%	23/329 (7.0%)	4.6%, 10.1%	38/592 (6.4%)	4.7%, 8.6%	0.522
**Hypertriglyceridemia**	**69/275 (25.1%)**	**20.2%, 30.5%**	**70/343 (20.4%)**	**16.4%, 24.9%**	**139/618 (22.5%)**	**19.3%, 25.9%**	**0.165**
Blood triglycerides ^a^ ≥ 150 mg/dL	62/248 (25.0%)	19.9%, 30.7%	60/303 (19.8%)	15.6%, 24.6%	122/551 (22.1%)	18.8%, 25.7%	0.144
Self-reported medication use for triglycerides	14/242 (5.8%)	3.4%, 9.3%	15/314 (4.8%)	2.8%, 7.6%	29/556 (5.2%)	3.6%, 7.3%	0.600
Self-reported elevated triglycerides diagnosis ^c^	32/242 (13.2%)	9.4%, 17.9%	27/312 (8.7%)	5.9%, 12.2%	59/554 (10.6%)	8.3%, 13.4%	0.089

Abbreviations: ♂, men; ♀, women; BMI, body mass index; CMD, cardiometabolic disease; DBP, diastolic blood pressure; DM2, type 2 diabetes mellitus; HbA1c, A1c hemoglobin; HDL, high-density lipoprotein; MetS, metabolic syndrome; NCEP-R ATP-III, National Cholesterol Education Program Adult Treatment Program, 3rd edition, revised criteria; SBP, systolic blood pressure. ^a^ Chi-squared test with 1 df for the comparison of prevalence rate estimates by natal sex. ^b^ Blood sample drawn while fasting. ^c^ Not an NCEP-R ATP-III criterion, but reported as a related study finding.

## Data Availability

The data supporting the study findings are not publicly available due to ethical and regulatory restrictions, as they contain sensitive clinical and social information from ALWH that could compromise participant confidentiality. De-identified, aggregate data and relevant study documentation (e.g., codebooks and analytic specifications) may be made available to qualified researchers upon reasonable request and with appropriate approvals, in accordance with institutional review board requirements and applicable data use agreements. Data requests should be directed to the corresponding author and Principal Investigator (H.L.C.).

## Supplementary Materials

The following supporting information can be downloaded at https://www.mdpi.com/article/10.3390/medsci14020200/s1, Supplementary Table S1. Instruments used in the CASI to assess Social Determinants of Health (SDoH) in the Colón C3 Study. Supplementary Table S2. Study variables from anthropometric and laboratory measurements. Supplementary Table S3. Summary of study procedures at each visit [69,70,71,72,73,74,75,76,77,78,79,80,81,82,83,84]. Supplementary Figure S1. Detail of the Colón Province, with the District of Colón in red. Supplementary Figure S2. Programmatic conceptual framework of the Colón C3 Study illustrating descriptive, analytic, and translational phases aligned with Specific Aims.

## Abbreviations

The following abbreviations are used in this manuscript:

ALWH	adults living with HIV
ART	antiretroviral therapy
AS	analytic sample
ASES	Adherence Self-Efficacy Scale
ASSIST-FC	Alcohol, Smoking and Substance Involvement Screening Test-Frequency and Concern
BMI	body mass index
CASI	computer-assisted self-interview
CI	confidence interval
CMD	cardiometabolic disease
DBP	diastolic blood pressure
DM2	type 2 diabetes mellitus
DSM-BIA	direct segmental multi-frequency bioelectrical impedance analyzer
DXA	dual X-ray absorptiometry
HbA1c	A1c hemoglobin
HDL	high-density lipoprotein cholesterol
HIV	human immunodeficiency virus
hsCRP	high-sensitivity C-reactive protein
IL	interleukin
INSTI	integrase strand transfer inhibitors
IQR	interquartile range
LDL	low-density lipoprotein cholesterol
LGBTQ+	lesbian, gay, bisexual, transgender, queer/questioning, and others
MetS	metabolic syndrome
MOS	Medical Outcomes Study
NCEP-R ATP-III	National Cholesterol Education Program Adult Treatment Program, 3rd edition, re-vised
PAB	Panamanian Balboa
PANES	Physical Activity Neighborhood Environment Scale
SBP	systolic blood pressure
SD	standard deviation
SDoH	social determinants of health
SSI	Substance-specific Involvement
TNF	tumor necrosis factor
UCLA	University of California-Los Angeles
UNAIDS	Joint United Nations Programme on HIV/AIDS
USD	United States Dollar
VLDL	very low–density lipoprotein cholesterol

## Appendix A. Aims, Methods, and Procedures for the Colón C3 Study

### Appendix A.1. Colón C3 Study Aims

The Colón C3 Study has three primary aims. This report focuses on the study’s first aim.

- Aim 1. Estimate the overall prevalence and 12-month trajectory of MetS per the National Cholesterol Education Program Adult Treatment Program, 3rd edition, revised (NCEP-R ATP-III) [59] and its five criteria (i.e., abdominal obesity, hypertriglyceridemia, reduced HDL cholesterol, hypertension, and hyperglycemia) among ALWH in Colón, Panama. We hypothesize that, compared to previously reported estimates for the general adult population, the prevalence rates and 12-month trajectories of MetS and its five diagnostic criteria will be higher among ALWH in Colón compared to the benchmark reported for Latin America.
- Aim 2. Describe and estimate the overall effect of therapeutic, laboratory, and socio-demographic variables on the prevalence and trajectory of MetS and its five diagnostic criteria in ALWH in Colón, Panama. We hypothesize that there is an effect modification on the prevalence and trajectory of MetS and its five diagnostic criteria by therapeutic (e.g., time on treatment, ART compliance, viral suppression), laboratory (e.g., levels of hsCRP and HbA1c), and socio-demographic (e.g., natal sex, age, gender, race, ethnicity, sexual orientation, employment/insurance status, substance use, time since HIV diagnosis) variables.
- Aim 3. Adapt clinical and preventative MetS guidelines to implement a local, pilot, clinical guideline for the assessment and treatment of MetS in ALWH in Colón, Panama, and evaluate its feasibility, acceptability, sustainability within the ART Clinic’s local capacity. We hypothesize that investigators, participants, and clinical providers will be engaged in the guideline development process and such a pilot protocol will be feasible, acceptable, and sustainable.

### Appendix A.2. Study Site

The Colón C3 Study was conducted at the ART Clinic located in Colón City, the capital of the homonym province in Panama (Supplementary Figure S1). The ART Clinic is overseen by the Ministry of Health and housed within the Dr. Manuel Amador Guerrero Hospital Complex. The ART Clinic provides comprehensive HIV care to an estimated active population of 844 ALWH as of 31 December 2024. The Colón province has a heterogeneous population estimated in 2025 at nearly 325,000 inhabitants [85], most of Afro-Caribbean descent. Indicators for the Colón province show significant socioeconomic disparities and the highest national mortality rates from CMD. Despite its economic importance due to the Colón Free Trade Zone and the Caribbean entrance to the Panama Canal, the province has a lower Human Development Index than the national average [86] (0.787 vs. 0.814) and faces persistent barriers to healthcare access [87]. These characteristics have a major impact in the local capacity to prevent, monitor, and treat the overlapping HIV and MetS epidemics in the province. In 2016, the ART Clinic achieved 100% linkage to care of adults recently diagnosed with HIV [88] and currently offers comprehensive services within the HIV care continuum.

### Appendix A.3. Ethical Registration, Oversight, and Reporting

This study was registered at the *Registro y Seguimiento de Investigación para la Salud* of Panama’s Ministry of Health (Registry No. 2722, dated 19 December 2019), approved and overseen by *Comité de Bioética de Investigación at Pacífica Salud* (Protocol No. 117, dated 18 March 2024) and by the University of Central Florida’s Institutional Review Board (STUDY00006622, dated 25 April 2024), all reviewed by the sponsor before participant enrollment (Fogarty International Center at the National Institutes of Health communication dated 1 May 2024).

Notwithstanding that the study procedures were deemed minimal risk for adverse events and serious adverse events (SAEs) and that the study was not designed to follow-up with participants who died during the study period, the *Comité de Bioética de Investigación* at Pacífica Salud, which operated as the IRB of record, requested the reporting of each death within 24 h of notification to the study personnel using the SAE form. Twelve deaths were notified by the Colón C3 Study to the IRB, all of which were considered SAEs not related to the study procedures. To advance the study’s research questions, the *Comité de Bioética de Investigación* at Pacífica Salud requested further investigation of these deaths. The 12 death certificates were requested to determine the point mortality rate by CMDs using an agreed-upon classification [89]. The death certificates reveal that 4 of 12 (33.3%) deaths were due to cardiometabolic events (2 acute myocardial infarctions, 1 stroke, 1 uncompensated diabetes with sepsis).

### Appendix A.4. Inclusion and Exclusion Criteria

Participants were eligible if they were adults ≥18 years; with a confirmed HIV diagnosis; actively receiving care at the Colón ART Clinic; and residents of the Colón province. Pregnancy was not an exclusion criterion, and 11 pregnant participants were recruited at baseline. Pregnancy was noted for separate direct segmental multi-frequency bioelectrical impedance analysis (DSM-BIA).

Participants were excluded from the DSM-BIA analyses if they had implanted electronic medical devices (e.g., pacemakers); severe open skin lesions on electrode contact points; of if unable to stand unassisted.

### Appendix A.5. Study Procedures

After site preparation, ethical approval, and validation of study procedures through an initial, pilot run with study staff and the first 20 participants enrolled, eligible participants were either approached by the study personnel or referred by the ART Clinic personnel. The study purpose and procedures were explained and eligible participants who showed interest underwent the informed consent process and received a unique participant code. Participants completed a computer-assisted self-interview (CASI) on a tablet device for approximately 30 min, with real-time secure online capture in REDCap (Vanderbilt University; Nashville, TN, USA). Study participants then underwent anthropometric measures by the study personnel and received a referral for fasting blood samples at the clinical laboratory. As a token of appreciation for their time, participants received a 5.00 PAB (1 PAB = 1 USD) prepaid mobile phone card at the completion of each study visit. Starting from the CASI administration, the study procedures were repeated every 6 ± 1 months for two additional visits.

### Appendix A.6. Study Variables and Instruments

Data sources were the CASI, anthropometric measurements, and blood test results. Supplementary Table S1 presents a summary of the SDoH instruments administered to the study participants. Briefly, all surveys in the CASI were in Spanish (all Cronbach’s α ≥ 0.77) and included 17 sociodemographic variables and 11 instruments (71 items) assessing SDoH from the *All of Us* Research Program methodology [59,90]. Two additional instruments administered were the HIV-treatment Adherence Self-Efficacy Scale (HIV-ASES [91]; 10 items Spanish version [92]) and the Alcohol, Smoking and Substance Involvement Screening Test-Frequency and Concern Items (ASSIST-FC [93]; 10 items Spanish version [94]).

Anthropometric data were collected by trained study personnel and included height to the nearest 0.01 m by mobile stadiometer (seca 213; seca gmbh & co; Hamburg, Germany), abdominal circumference to the nearest 0.01 m by digital measuring tape (Health-o-meter HDTM012-69; Sunbeam Products, Inc.; Boca Raton, FL, USA), blood pressure to the nearest 1 mmHg by aneroid sphygmomanometry (Prestige model S79; Prestige Medical; Dublin, Ireland), and total and DSM-BIA (InBody 270; InBody Co., Cerritos, CA, USA) using 8-point tactile electrodes (thumbs and palms; heels and forefeet) to measure impedance across 5 segments (right/left arms, trunk, right/left legs) at multiple frequencies (commonly 20 and 100 kHz). This allows for the generation of estimates of body water (intracellular/extracellular), dry lean mass, skeletal muscle mass, and body fat mass/percentage without applying population-specific empirical equations during measurement [35].

All blood samples were collected at the ART Clinic’s local laboratory. One-time laboratory tests included glycated hemoglobin A1c (HbA1c), high-sensitivity C-reactive protein (hsCRP), CD4+ cell count, and viral load. Fasting (8–12 h) laboratory tests repeated at every study visit included blood glucose, triglycerides, high-, low-, and very low–density lipoprotein (HDL, LDL, and VLDL, respectively) and total cholesterol. Most analyses were conducted at the ART Clinic’s local laboratory in Colón City and, in the case of CD4+ cell counts and viral loads by real-time polymerase chain reaction, analyses were conducted at the central public-health reference laboratory in Panama City. Participants who did not complete the laboratory tests as scheduled in their ART Clinic’s regular visits received follow-up reminders from both the ART Clinic and the study personnel. To facilitate access to and compliance with laboratory testing, the ART Clinic, the local laboratory, and the study personnel extended the regular operation hours (07:00–15:00 h Mondays–Fridays) to include early and late opening and weekends, for a total of 63 days with extended operations. Laboratory test results were reported both for the study database and for the participants’ charts at the ART Clinic. The detailed list of anthropometric and laboratory variables is provided in Supplementary Tables S2 and S3 summarizes the study procedures at every study visit. Supplementary Figure S2 presents the analytic framework for each aim of the Colón C3 Study.
