# Supplementary material for: Cardiometabolic Status of Adults Living with HIV in Panama—Baseline Results of the Colón C3 Study
Source: Med Sci (Basel). 2026 Apr 15;14(2):200. doi: 10.3390/medsci14020200 (PMC13108132; doi:10.3390/medsci14020200)
Supplement: Supplementary file 1 [file medsci-14-00200-s001.zip › medsci-4209766-supplementary.pdf]

Table S1. Instruments used in the CASI to assess Social Determinants of Health (SDoH) in the Colón C3 Study.

Instrument	Construct Measured	Items: Total	Items: Min Valid	Cronbach's α: English	Cronbach's α: Spanish	Range of Responses	Scoring Method	Possible Score Range	Interpretation of Higher Scores	Sample Item
**Domain: Social and Community Context**										
Social Cohesion Neighborhood Scale [69]	Neighborhood social organization	4	4	0.74	NR	1=Strongly agree to 5=Strongly disagree	Mean of 4 items	1–5	Lower cohesion	People in this neighborhood can be trusted
mMOS Social Support [70], Total Scale) Spanish [71])	Perceived availability of support from others when needed	8	≥6	0.93	0.91	1=None to 5=All of the time	Average of items	1–5	Greater perceived availability of support from others when needed. Indicates stronger overall social network	How often do you have someone you can rely on for help when you need it?
mMOS Social Support [70], Instrumental Support Subscale	Perceived access to practical help	4	≥3	NR	NR	1=None to 5=All of the time	Average of items	1–5	More perceived access to practical help (e.g., with chores or transportation)	Someone to help you if you were confined to bed
mMOS Social Support [70], Emotional Support Subscale	Perceived access to emotional comfort	4	≥3	NR	NR	1=None to 5=All of the time	Average of items	1–5	More perceived access to emotional comfort (e.g., feeling loved, having someone to talk to)	Someone you can count on to listen to you when you need to talk
UCLA Loneliness Scale (ULS-8) [72]	Perceived loneliness	8	≥6	0.84	NR	1=Never to 4=Often	Mean of items	1–4	Greater feelings of loneliness, isolation, and lack of social connectedness	I feel isolated from others
Everyday Discrimination Scale [73] (Spanish [74])	Perceived unfair treatment in daily life	9	≥7	0.88	0.83	0=Never to 5=Almost every day	Mean of items	0–5	More frequent experiences of perceived unfair treatment in daily life. Reflects higher psychosocial stress and social marginalization	You are treated with less courtesy than other people
Perceived Stress Scale (PSS-10) [75] (Spanish [76, 77])	Perceived stress	10	≥8	0.78	0.81–0.95	1=Never to 5=Very often	Item mean ×10	10–50	Greater perceived stress in the past month, reflecting feeling	In the last month, how often have you felt

									overwhelmed or unable to cope	nervous and stressed?
Domain: Neighborhood and Built-in Environment										
Neighborhood Disorder Scale, Total [78]	Perceived physical and social neighborhood disorder	13	≥12	0.92	NR	1=Strongly disagree to 4=Strongly agree	Average of items	1–4	More neighborhood disorder	There is a lot of graffiti in my neighborhood
Neighborhood Disorder Scale [78], Physical Disorder Subscale	Neighborhood physical disorder	6	≥5	0.84	NR	1=Strongly disagree to 4=Strongly agree	Mean of items, reverse code items 5 and 6	1–4[a]	Greater visible disorder (e.g., litter, vandalism, noise), suggesting neighborhood neglect or distress	There is litter, broken glass, or trash on the sidewalks and streets
Neighborhood Disorder Scale [78], Social Disorder Subscale	Neighborhood social disorder	7	≥6	0.87	NR	1=Strongly disagree to 4=Strongly agree	Mean of items, reverse code items 12 and 13	1–4[a]	More perceived threats to safety and social conflict (e.g., crime, loitering, public intoxication)	People in your neighborhood are drinking alcohol or using drugs in public
PANES [79] – Walking and Bicycling Subscale	Perceived neighborhood walkability	5	≥4	0.78	NR	1=Strongly disagree to 4=Strongly agree	Average of items	1–4[a]	Better walkability	There are sidewalks on most streets in my neighborhood
PANES [79] – Crime Safety Subscale	Perceived neighborhood safety	2	2	NR	NR	1=Strongly disagree to 4=Strongly agree	Average of items	1–4[a]	Safer neighborhood	I feel safe walking in my neighborhood during the day
PANES [79] – Residential Density Subscale	Neighborhood housing type	1	1	NR	NR	Categorical (5 options)	Single item	N/A	Higher density	What is the main type of housing in your neighborhood?
Domain: Economic Stability										
Children's HealthWatch Hunger Vital Sign [80]	Household food security	2	2	NR	NR	Often true–Sometimes true–Never true	Binary classification	Secure/ Insecure	Insecure if any item ≠ Never true	Within the past 12 mo, we worried whether our food would run out before we got money to buy more

Housing Instability [81]	Residential mobility	1	1	NR	NR	Number of moves in past 12 mo	Single item	Numeric	More instability	In the last 12 mo, how many times have you or your family moved from one home to another?
Housing Quality [82]	Housing problems	8	—	NR	NR	Check all that apply	Binary (any problem vs none)	0–1	Housing problem present	Think about the place you live. Do you have problems with any of the following? (e.g., mold, water leaks)
Domain: Health and Healthcare										
Everyday Discrimination Scale in Medical Settings [83] (Spanish [84])	Healthcare discrimination	7	≥5	0.89	≥0.80	1=Never to 5=Always	Average of items	1–5	More perceived discrimination	You receive poorer service than others in health care settings
HIV-ASES [91] (Spanish [92])	Confidence in ability to adhere to HIV treatment plans (ART, nutrition, exercise) in the face of barriers	12	—	NR[b]	NR	1=Cannot do it at all to 10=Certain can do it	Average of items	1–5	Greater adherence self-efficacy	Stick to your treatment plan even when side effects interfere with daily activities
ASSIST-FC, Substance Specific Involvement (SSI) [93], (Spanish [94])	Risk level of substance use based on frequency of use and concern expressed by others (brief screener for hazardous/harmful use)	2	—	NR[c]	NR	Frequency: 0=Never; 2=Once or twice; 3=Monthly; 4=Weekly; 6=Daily/almost daily. Concern: 0=No, never; 6=Yes, in past 3 mo; 3=Yes, but not in past 3 mo	Sum Q1+Q2 per substance; classify risk (Alcohol: 0–5 low, 6–8 moderate*, 9–12 high; Other substances: 0 low, 2–6 moderate, 7–12 high). *or if heavy drinking threshold is met	0–12 per substance	Greater risk for hazardous/harmful substance use. Indicates need for brief intervention or further assessment	Has anyone ever expressed concern about your use of [substance]?

Abbreviations: ASSIST-FC, Alcohol, Smoking and Substance Involvement Screening Test-Frequency and Concern; CASI, computer-assisted self-interview; HIV-ASES, HIV treatment Adherence Self-Efficacy Scale; min, minimum; mMOS, Modified Medical Outcomes Study; NR, not reported; PANES, Physical Activity and Neighborhood Environment Scale[82]; UCLA, University of California-Los Angeles.

[a]Modified to a 5-item Likert scale to match the rest of the scales.

[b]The source publication [66] reports high internal consistency coefficients (ρ>0.90) and test–retest reliability (3 month r>0.70; 15 month r>0.40), but not Cronbach's α.

[c]The source publication [68] is a psychometric reduction/validation study; internal consistency (Cronbach's α) is not the preferred metric for this 2 item classifier, and the article emphasizes sensitivity/specificity and comparative validity vs. the full ASSIST.

Table S2. Study variables from anthropometric and laboratory[a] measurements.

Variable	Units	Reference Range	Measurement Method	Purpose in Study	Interpretation of Higher Value
Height	m	N/A	Stadiometer, standing	Assess stature, compute BMI	Taller stature
Weight	kg	N/A	InBody	Assess body weight, compute BMI	Greater body mass
BMI	kg/m²	<18.5 underweight 18.5–24.9 normal ≥25 overweight ≥30 obese	Calculated (kg/m²)	Assess obesity status	Greater obesity risk
Waist Circumference	cm	>102 (men) >88 (women)	Digital tape at midpoint between iliac crest and rib	Assess abdominal obesity (MetS criterion)	Greater visceral fat
Blood Pressure	mmHg	≥130/85	Digital sphygmomanometer, seated	Assess hypertension risk (MetS criterion)	Greater cardiovascular risk
Glucose	mg/dL	≥110	Venipuncture, fasting ≥8h	Assess glycemic control (MetS criterion)	Greater risk for DM2
HbA1c	%	<5.7 normal 5.7–6.4 prediabetes ≥6.5 diabetes	Venipuncture, fasting ≥8h	Assess chronic glycemia	Poorer glycemic control
Triglycerides	mg/dL	<150	Venipuncture, fasting ≥8h	Assess dyslipidemia (MetS criterion)	Greater CMD risk
HDL Cholesterol	mg/dL	>40 (men) >50 (women)	Venipuncture, fasting ≥8h	Assess protective lipid fraction (MetS criterion)	Lower CMD risk
LDL Cholesterol	mg/dL	<100 optimal; ≥160 high	Venipuncture, fasting ≥8h	Assess atherogenic lipid fraction	Greater CMD risk
Total Cholesterol	mg/dL	<200 desirable; ≥240 high	Venipuncture, fasting ≥8h	Assess overall lipid status	Greater CMD risk
hsCRP	mg/L	<1 low; 1–3 moderate; >3 high	Venipuncture, fasting ≥8h	Assess systemic inflammation	Greater inflammation and CMD risk
CD4+ Count	cells/mm³	>500 normal; <200 severe immunosuppression	Venipuncture, fasting ≥8h, flow cytometry	Assess HIV immune status	Better immune function
HIV Viral Load	copies/mL	≤40 undetectable	Venipuncture, fasting ≥8h, PCR	Assess ART effectiveness	Poorer viral suppression

Abbreviations: BMI, body mass index; CMD, cardiometabolic disease; DM2, type 2 diabetes mellitus; MetS, metabolic syndrome; PCR, polymerase chain reaction.
[a]All laboratory samples were collected after a minimum 8-hour fasting period.

Table S3. Summary of study procedures at each visit.

Study Procedures	Visit 1	Visit 2 (6 ± 1 mo)	Visit 3 (12 ± 1 mo)
Informed Consent	●		
CASI			
Demographics	●	●	●
MetS by self-report	●[a]	●	●
SDoH	●	○	○
HIV-ASES	●	●	●
ASSIST-FC	●	●	●
Anthropometry			
Height	●	●	●
Weight	●	●	●
Abdominal circumference	●	●	●
Blood pressure	●	●	●
DSM-BIA			
Laboratory samples[b]	●	●	●
Cholesterol (HDL, LDL, VLDL, total)	●	●	●
Triglycerides	●	●	●
Glucose	●	●	●
HbA1c	●	○	○
hsCRP	●	○	○
Viral load	●	○	○
Height	●	○	○
CD4+ lymphocyte count	●	○	○

Abbreviations: ASSIST-FC, Alcohol, Smoking and Substance Involvement Screening Test-Frequency and Concern [68]; CASI, computer-assisted self-interview; DSM-BIA, direct segmental multi-frequency bioelectrical impedance analysis; HbA1c, glycated hemoglobin; HDL, high-density lipoprotein; HIV-ASES, HIV treatment Adherence Self-Efficacy Scale [66]; hsCRP, high-sensitivity C-reactive protein; LDL, low-density lipoprotein; MetS, metabolic syndrome; mo, month; SDoH, social determinants of health; VLDL, very low-density lipoprotein.

[a]Due to a glitch in the CASI skip logic, this instrument was not displayed to participants during Visit 1.

[b]All laboratory samples were collected after a minimum 8-hour fasting period.

● Procedure conducted at the study visit.

○ Procedure conducted at the study visit if not completed during the prior visit.

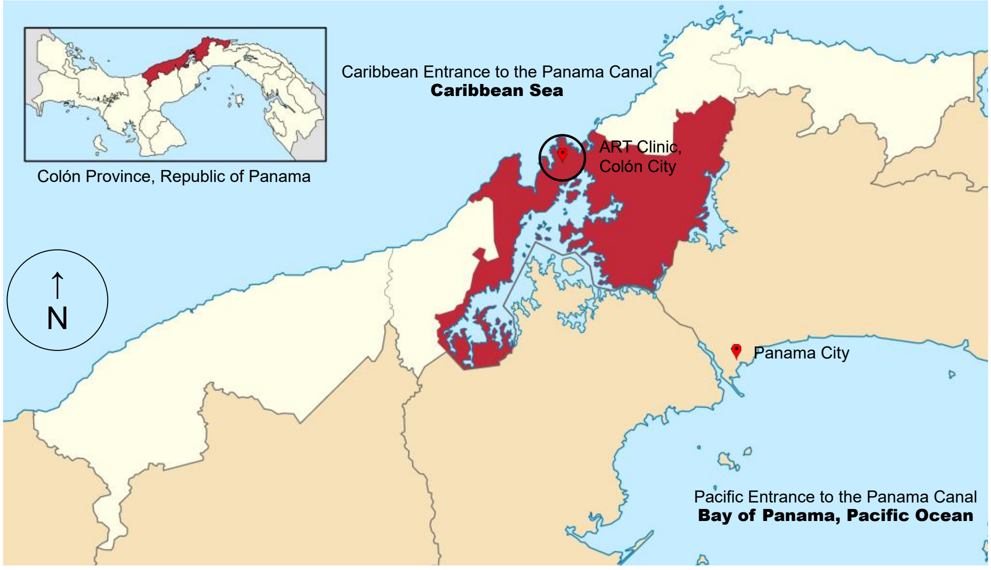

Figure S1. Detail of the Colón Province, with the District of Colón in red.

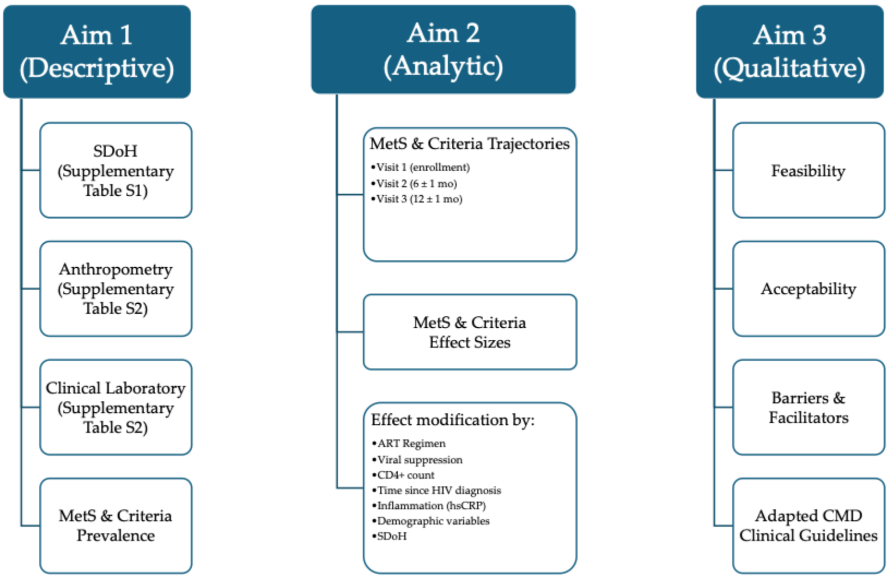

Figure S2. Programmatic conceptual framework of the Colón C3 Study illustrating descriptive, analytic, and translational phases aligned with Specific Aims.
